# Photosynthesis 2.0

**DOI:** 10.3390/ijms24054355

**Published:** 2023-02-22

**Authors:** Stefano Cazzaniga, Matteo Ballottari

**Affiliations:** Department of Biotechnology, University of Verona, 37134 Verona, Italy

Photosynthesis is a process that provides the continuous income of energy needed to sustain life on our planet. The main group of photosynthetic organisms uses water as an electron donor generating oxygen in the process. Many life forms depend upon oxygenic photosynthetic organisms to generate chemical energy from light, to extract oxygen from water, and to regulate CO_2_ concentrations in the atmosphere. During the last decade, several advancements have been made surrounding the fundamental molecular mechanisms at the base of photosynthesis; the study of structures of PSII, PSI complexes, and antenna proteins; how they interact and are organized inside the thylakoid membrane; and how they respond to different light regimes and to excess light stress. However, there are still several gaps in our understanding of the photosynthetic processes that need to be filled by advanced and multidisciplinary research efforts. An understanding of the mechanisms, constrains, and limitations of photosynthesis will allow for the design and testing of a synthetic biology approach that could improve the efficiency of light conversion toward Photosynthesis 2.0, with enhanced carbon fixation and biomass/biomolecule production. This understanding could help to fill the gap between actual photosynthetic productivity and the need to manage the forecasted increase in the global population. With limited agricultural land and increasing human population, it is essential to enhance productivity, and increasing the photosynthesis yield of plant and algae offers the highest potential for trying to solve these critical issues. This Special Issue aims to add to this effort by trying to provide new insight on different aspects of the photosynthesis. The papers presented in this Special Issue are studied and described below:

(i) a new fluorescence technique to determine the aggregation state and quenching capacity of antenna protein applied to a new microalgal strain;
(ii) a pigment composition and photosynthetic response in a poplar cultivar with increased anthocyanin content;
(iii) the role of the TOR kinase in regulating photosynthesis and photomorphogenesis in plant;
(iv) the change in photosynthetic apparatus and photosynthetic yield in wheat regarding pathogen infection.

PSII and PSI are the complexes responsible for trapping light energy and converting it in chemical energy. The two photosystems have a similar organization being composed by a core complex surrounded by an array of antennae [1,2]. While antenna proteins increase the light-harvesting efficiency in low-light conditions, too many excitations are transferred to the photochemistry in excessive light conditions and the electron transport chain becomes saturated, increasing reactive oxygen species formation and photoinhibition [3,4]. Non-photochemical quenching (NPQ) dissipates the excess light energy absorbed by photosystems as heat in order to decrease the amount of excited chlorophylls [5]. NPQ is a complex mechanism, not completely elucidated, that involves LHCII antenna aggregation [6]. Crepin and co-workers [7] describe a new approach using fluorescence correlation spectroscopy (FCS) to study the in vitro clustering of plant LHCII antennas in a surfactant solution. This technique allows the LHCII oligomerization state and their fluorescence properties to be simultaneously estimated in very small volumes and can help to provide new details on the molecular mechanisms of LHCII aggregation and contribute to their quenching. The same technique was used to study the aggregation state of antenna particles, isolated from alveolate alga *Chromera velia* [8], which involved comparing their sizes and fluorescence intensities with the monomeric and trimeric antennas from higher plants. The *C. velia* antenna complexes, called Chromera Light Harvesting (CLH), arecloser to LHC from dinoflagellate and diatoms, but with unique features such as red-shifted antenna proteins accumulated in red light. Little information was available on the organization of the LHC in this species and FCS was able to distinguish between antennae in the monomeric state and the trimeric state. CLH antennae resulted in monomeric state and these monomers were found in a more quenched state than plant trimeric LHCII. These data were also confirmed by biochemical analysis. The FCS technique, described in these two papers, could help to obtain data in the organization and quenching capacity of the photosynthetic complexes of different species. 

Poplar is a fast-growing tree species and an important source of raw materials with a high economic value. This species is used as a model for research on the physiological and molecular mechanisms of trees. Wang and co-workers [9] characterized a Populus deltoides cultivar (ZHP) that constitutively accumulates a higher amount of anthocyanins, given that the leaves have a darker reddish color. The higher anthocyanin content in the ZHP reduces the light-harvesting capacity, resulting in a decrease in photosynthetic efficiency. 

The target of rapamycin (TOR) kinase is a central component of nutrient sensing in all eukaryotic organisms and it is also fundamental for plant development [10]. TOR activity modulates many cellular and metabolic processes, including nutrient assimilation and transport, cell division and expansion, and plant development [11]. Compared to the case of mammalians or yeasts, the understanding of plant and algal TOR is still limited. Song and co-workers [12] highlighted the role of TOR on regulating photosynthesis and, in particular, on sensing the nutrients produced by photosynthesis, as well as regulating light absorption and chloroplast development. Authors described TOR cascade signaling in photomorphogenesis in the plant transition from dark to light. They also described the role of this kinase in chloroplast biogenesis and maturation, as well as the biosynthetic pathway of chlorophylls.

Photosynthesis is very sensitive to abiotic stress [13,14]. Drought, high or low temperatures, and excess irradiances may lead to a rapid decline in photosynthetic efficiency [15]. Indeed, photosynthesis could be used as an early sensing tool to detect these types of stresses. In addition, photosynthesis changes in response to biotic stress in relation to various insects and pathogens [16]. Yang and co-workers described the photosynthetic changes in wheat in response to fungal pathogens [17]. Available literature data were integrated to highlight the photosynthetic changes in the early and late stages of pathogen infection. Photosynthetic changes during the early stage of infection are positively correlated with the resistance ability of plants, while changes in the late stage result from the resistance response itself. This correlation can also be attributed to ROS accumulation, a signaling molecules can be used to activate protective responses. Photosynthesis can thus be a useful indicator for interactions between hosts and pathogens.

## Data Availability

Not applicable.
